# Analysis of extraocular muscle volumes in idiopathic hypertrophic pachymeningitis patients

**DOI:** 10.1371/journal.pone.0309638

**Published:** 2025-04-29

**Authors:** Suppakul Kitkamolwat, Supichaya Soonthornpusit, Akarawit Eiamsamarng, Natthapon Rattanathamsakul, Niphon Chirapapaisan, Chanon Ngamsombat

**Affiliations:** 1 Department of Radiology, Faculty of Medicine Siriraj Hospital, Mahidol University, Bangkok, Thailand; 2 Department of Ophthalmology, Faculty of Medicine Siriraj Hospital, Mahidol University, Bangkok, Thailand; 3 Department of Medicine, Faculty of Medicine Siriraj Hospital, Mahidol University, Bangkok, Thailand; UCSF: University of California San Francisco, UNITED STATES OF AMERICA

## Abstract

**Background:**

Idiopathic hypertrophic pachymeningitis (HP) is a rare chronic inflammatory condition without an identifiable cause characterized by fibrous thickening of the dura mater, which can involve the extraocular muscles (EOM).

**Objective:**

To evaluate volumetric changes of EOM in idiopathic HP patients compared with healthy controls (HC) and study the correlation with ocular motility disturbance.

**Materials and method:**

Twenty-two idiopathic HP patients diagnosed and underwent 3T MRI between 2017 to 2021 at Siriraj Hospital and 22 age- and sex-matched HC were included in this retrospective study. EOM was manually segmented from the T1W image using 3D Slicer software, and volume was calculated using FSL software. T-tests and Mann-Whitney U tests were used to compare EOM volumes between the idiopathic HP and control groups. Pearson’s correlation coefficient was then used to assess the correlation between ocular motility and EOM enlargement.

**Results:**

In idiopathic HP patients, the average EOM volumes, including the medial rectus (p = 0.002 each), inferior rectus (right p = 0.08, left p < 0.01), inferior oblique (right p = 0.009, left p = 0.005), right lateral rectus (p = 0.005), right superior oblique (p = 0.004), and left superior rectus (p = 0.005) muscles, were significantly larger compared to those in HC, particularly in the left IR and both MR. However, there was no significant correlation between the enlargement of these 9 EOMs and the extraocular movement limitation.

**Conclusion:**

In idiopathic HP patients, significantly larger EOM volumes were found compared to control subjects. This enlargement could be due to the diffuse infiltrative histopathology potentially involving microstructures in the EOM. Extraocular movement limitations may be related to cranial nerve involvement. However, the enlarged EOM volumes show no significant correlation with extraocular movement limitation.

## 1. Introduction

Hypertrophic pachymeningitis (HP) is a rare chronic inflammatory condition characterized by fibrous thickening of the cerebral and/or spinal dura mater [1].HP is classified into two types based on its etiology: primary (idiopathic) HP, which has no identifiable cause, and secondary HP, which includes coexisting causes such as infectious (such as tuberculosis, syphilis, and sarcoidosis), autoimmune (such as IgG4-related disease), vasculitis (such as Wegener’s granulomatosis), and neoplastic (such as lymphoma and metastasis) [2,3].

HP prevalence has been reported as 0.949/100,000 persons based on the Japanese population, which idiopathic HP is about half [1–3]. The pathophysiology of idiopathic HP is currently unclear. Histopathological examination reveals non-specific chronic inflammatory changes in the dura, including extensive fibrosis and often-detected inflammatory cell infiltration. The dura mater surface may contain small, mature lymphocytes, plasma cells, and epithelioid histiocytes [4].

Idiopathic HP is diagnosed based on clinical symptoms such as headaches, diplopia, visual loss, ptosis, and facial pain, with thickening and enhancement of the dura mater seen on MRI imaging and/or dura mater biopsy, which is the gold standard of diagnosis [3].

Previous studies of HP have characterized clinical presentation, laboratory findings, and neuroradiologic features [1–3]. Clinically, they are varying based on the location of inflammatory structures. Diplopia is the most prevalent clinical manifestation, reported in 85.2% of cases [3] and being a potential consequence of extraocular movement limitation, which can result from either neurological or myopathic processes [5,6]. While a direct evaluation of neuronal control mechanism is not feasible, structural extraocular muscle (EOM) change is more easily observed. However, the details of EOM damage in this disease remains limited.

The aim of our study was to evaluate the volumetric changes of all EOM in idiopathic HP patients compared to age-matched, sex-matched healthy controls (HC) and to study the correlation with ocular motility disturbance. Our hypothesis was that the abnormal EOM volumes reflect degree of EOM limitation.

## 2. Materials and methods

### Participants

This retrospective study included twenty-two idiopathic HP patients diagnosed and underwent 3T MRI between 2017–2021 in the ophthalmology and neurology department at Siriraj Hospital. The study fully complied with the Declaration of Helsinki. Patient informed consent was waived by the ethics committee. The Siriraj Institutional Review Board approved the study (certificate of approval number Si 055/2023).

The entry criteria were defined as follows: 1) The diagnosis was based on dura mater biopsy or dura mater thickening or enhancement on MRI imaging, and 2) dural thickening or enhancement could not be explained by intracranial hypotension, neoplastic pachymeningitis, or other coexisting conditions [3]. Patients with a history of other etiologies affecting EOMs, such as prior brain surgery or trauma, underlying brain tumor or brain metastasis, focal mass at the EOM, or any EOM enlargement were excluded. Twenty-two age-matched and sex-matched healthy controls were recruited for MRI evaluation only.

### Ophthalmological examination

The data was gathered from medical records evaluated by neuro-ophthalmologist at Siriraj Hospital. The EOM function was evaluated by asking the patient to move their eyes in an H-shaped pattern. The ophthalmic examination was performed by two neuro-ophthalmologists and a third neuro-ophthalmologist adjudicated the discrepancies.

### MRI image acquisition

All patients were scanned using clinical MRI scanners (Signa 3.0T (General Electric Healthcare, USA), MAGNETOM Vida 3.0T (Siemens Healthcare, Germany), and Archieva 3.0T (Philips Healthcare, Netherlands)) at Siriraj Hospital between 2017–2021 with a 32-channel head coil. The MRI protocol included a T1W image sequence using three-dimensional TFE with the following parameters: echo time (TE) = 4.61 ms, repetition time (TR) = 9.53 ms, matrix size = 352 x 352, field-of-view (FOV) = 230 x 230 x 172 mm³, voxel size = 0.72 x 0.72 x 0.5 mm³, flip angle = 8°, scan time 6 minutes.

### Data processing

In brief, all EOM raw data were collected as DICOM images and transformed to NiFTI format using DICOM to NiFTI software.

Segmentation of the EOM was performed using 3D Slicer software v.5.6.1. The region of interests (ROI) of the EOMs were drawn manually by two radiologists (S.K. and C.N., with 3 and 15 years of experience, respectively), and finalized by consensus.

**Fig 1:**
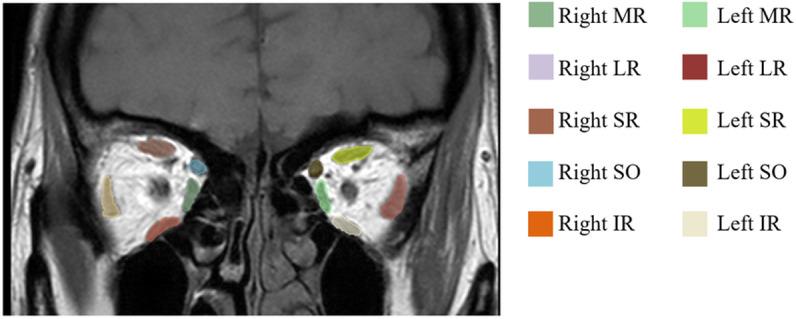
MRI T1 weighted images in coronal plane of a representative case with EOM segmentation by using 3D slicer software.

The medial rectus (MR), inferior rectus (IR), lateral rectus (LR), superior oblique (SO), and inferior oblique (IO) muscles were segmented separately in the coronal plane of the T1W image (Fig 1). The superior rectus (SR) and levator palpebrae superioris muscles were measured together due to such a close relationship that are difficult to delineate the outline and referred to as the superior rectus muscle [7–10].

The calculation of each EOM volume from the outlined ROI was performed using FSL Software (https://fsl.fmrib.ox.ac.uk).

## 3. Statistical analysis

After the image processing was done, statistical analysis was performed using SPSS (PASW Statistics for Windows, version 18.0).

Demographic data, including age, sex, and duration between eye and MRI examinations, were reported as means and standard deviations (SD). The quantitative data of the subjects with idiopathic HP and the healthy control group were compared using T-tests. The categorical factor of the subjects with idiopathic HP and the healthy control group was compared using the Chi-square test. Categorical data, including the location of dural thickening, were reported as frequency and percentage.

Comparisons of EOM volumes between patients and control groups were performed using T-tests for normal distributions and the Mann-Whitney U test for non-normal distributions. Pearson’s correlation was used to identify a significant correlation between EOM volume and ocular motility. The criteria for statistical significance were defined as a p-value < 0.05.

## 4. Results

### Clinical description

Clinical and demographic data for the idiopathic HP patients and HC are presented in Table 1. Twenty-two idiopathic HP patients, including 8 males and 14 females (mean age 51.50 ± 15.41 years), and 22 healthy controls in the sex-matched and age-matched groups (mean age 51.41 ± 15.30 years) were included for analysis. There is no statistical difference in age or sex between the idiopathic HP and control groups (p = 0.984 and p = 1.000, respectively). The mean duration between the date of eye examination and MRI examination is 59.64 ± 99.40 days.

**Table 1:** Demographic data.

	Group, n (%)		P-value
	Idiopathic HP (n = 22)	Healthy control (n = 22)	
Age (mean ± sd) (years)	51.50 ± 15.41	51.41 ± 15.30	0.984
Sex			1.000
Male	8 (36.4)	8 (36.4)	
Female	14 (63.6)	14 (63.6)	
Duration between eye examination and MRI imaging (mean ± sd) (days)	59.64 ± 99.40	–	

In idiopathic HP patients, most of the dural thickening is involved in the cavernous sinus and tentorium cerebelli (each 11/22 patients; 50%).

### MRI imaging analysis

The total 12 EOM volumes from both eyes of idiopathic HP patients and healthy control groups were measured and summarized in Table 2.

**Table 2:** Statistic data compared the average extraocular muscle volume (mm^3^) of idiopathic HP patients versus healthy control patients.

Extraocular muscle	EOM volume ($mm^{3}$)		P-value
	Idiopathic HP;mean ± SD	Healthy control ;mean ± SD	
Right Medial rectus (MR)	560.65 ± 122.89	445.47 ± 111.97	0.002*
Right Lateral rectus (LR)	624.03 ± 131.76	532.82 ± 99.77	0.005*
Right Superior rectus (SR)	509.63 ± 154.99	438.97 ± 118.05	0.096
Right Superior oblique (SO)	340.71 ± 70.15	271.07 ± 81.23	0.004*
Right Inferior rectus (IR)	496.78 ± 134.01	403.97 ± 82.60	0.008*
Right Inferior oblique (IO)	300.31 ± 117.56	217.45 ± 79.83	0.009*
Left Medial rectus (MR)	554.06 ± 138.19	436.26 ± 100.66	0.002*
Left Lateral rectus (LR)	599.05 ± 145.34	505.53 ± 130.41	0.054
Left Superior rectus (SR)	533.22 ± 146.82	418.74 ± 108.25	0.005*
Left Superior oblique (SO)	315.33 ± 99.93	267.05 ± 78.92	0.083
Left Inferior rectus (IR)	512.07 ± 98.18	398.00 ± 69.46	<0.001*
Left Inferior oblique (IO)	282.52 ± 82.17	215.74 ± 67.51	0.005*

* denotes significant p-value

**Fig 2:**
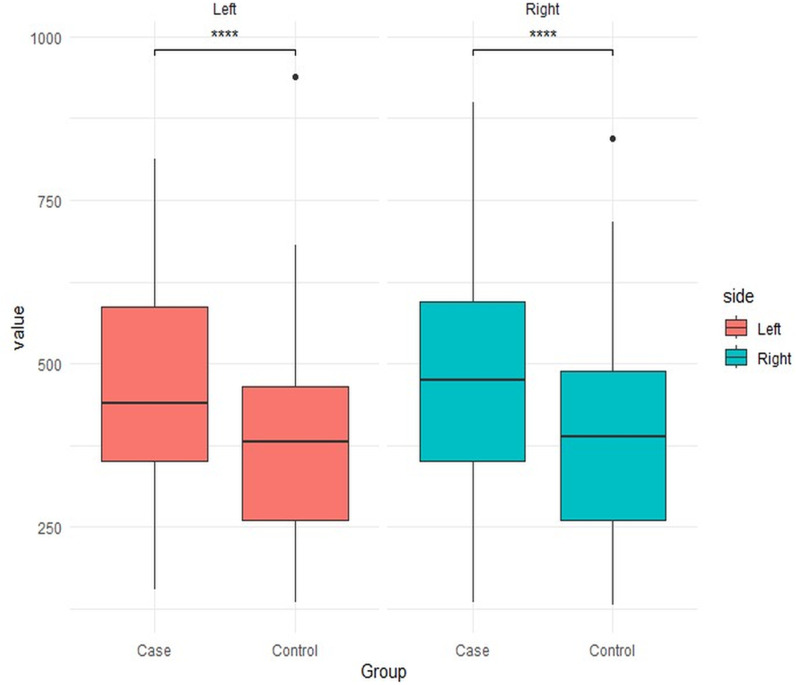
Box plots comparison of the extraocular muscle volume (mm^3^) of left and right eyes between idiopathic HP patients and healthy controls.

In brief, the average EOM volumes (mm³) in the left eye of idiopathic HP patients were 466.046 ± 170.261 mm³, and in the right eye were 472.022 ± 168.304 mm³, while the control group had 373.557 ± 137.288 mm³ and 384.296 ± 144.018 mm³. The average EOM volumes of both the left and right eyes were significantly greater than those of the control group (p ≤ 0.0001). (Fig 2)

For each EOM, the mean volumes in idiopathic HP patients were 560.65 ± 122.89 mm³ in the right medial rectus, 624.03 ± 131.76 mm³ in the right lateral rectus, 509.63 ± 154.99 mm³ in the right superior rectus, 340.71 ± 70.15 mm³ in the right superior oblique, 496.78 ± 134.01 mm³ in the right inferior rectus, 300.31 ± 117.56 mm³ in the right inferior oblique, 554.06 ± 138.19 mm³ in the left medial rectus, 599.05 ± 145.34 mm³ in the left lateral rectus, 533.22 ± 146.82 mm³ in the left superior rectus, 315.33 ± 99.93 mm³ in the left superior oblique, 512.07 ± 98.18 mm³ in the left inferior rectus, and 282.52 ± 82.17 mm³ in the left inferior oblique muscles.

In comparison to age-matched and sex-matched healthy controls, 9 of 12 EOM muscles showed significantly larger volumes. These included both MR (each p = 0.002), both IR (right p = 0.08 and left p < 0.01), both IO (right p = 0.009 and left p = 0.005), right LR (p = 0.005), right SO (p = 0.004), and left SR muscles (p = 0.005). However, there was no statistically significant difference in the volume of the right SR, left LR, and left SO muscles (p = 0.096, p = 0.054, and p = 0.083, respectively).

### Relationship between clinical and MRI measures

Correlation with ocular function was assessed only in 9 of the 12 EOM (both MR, both IR, both IO, right LR, right SO, and left SR), which had statistically significant enlargement from prior analysis. Using Pearson’s correlation, there was no statistically significant correlation between all 9 enlarged EOM and ocular motility in idiopathic HP patients, as presented in Table 3.

**Table 3:** Correlation between the significant enlarged EOM volumes (mm3) of idiopathic HP patients and the ocular motility.

Extraocular muscle	Mean volume in HP ($mm^{3}$), Mean ± sd	Percentage of ocular motility limitation, Mean ± sd	Pearson Correlation(P-value)
			EOM volume vs percentage of ocular motility limitation
Right IO	300.31 ± 117.56	11.59 ± 24.17	−0.142(0.527)
Right IR	496.78 ± 134.01	11.59 ± 24.94	−0.337(0.125)
Right SO	340.71 ± 70.15	11.59 ± 24.94	0.051(0.821)
Right MR	560.65 ± 122.89	7.50 ± 21.69	−0.013(0.955)
Right LR	624.03 ± 131.76	26.14 ± 33.37	0.249(0.264)
Left SR	533.22 ± 146.82	3.18 ± 9.45	−0.107(0.637)
Left IO	282.52 ± 82.17	4.55 ± 13.35	0.180(0.423)
Left IR	512.07 ± 98.18	3.18 ± 9.45	−0.221(0.324)
Left MR	554.06 ± 138.19	4.55 ± 11.84	−0.321(0.145)

A p-value < 0.05 indicates statistically significance

## 5. Discussion

Hypertrophic pachymeningitis (HP) is a rare fibrosing inflammatory disorder characterized by thickening of the dura mater, either focal or diffuse. The diagnosis of idiopathic HP is made by excluding various infectious, autoimmune, and neoplastic diseases. Therefore, when the clinical and imaging findings are consistent, a comprehensive investigation should be conducted to rule out any underlying pathological causes for the condition. Clinicals vary based on the location of inflammatory structures included diplopia, one of the consequence of EOM limitation.

Histopathological examination reveals non-specific inflammatory change in dura devoid of granulomas, giant cells or epithelioid cells which frequently characterized by dense fibrosis and inflammatory cell infiltration including small mature lymphocytes, plasma cells and epithelioid cells [11]. In our study, we found that 9 out of 12 EOMs (both MR, both IR, both IO, right LR, right SO, and left SR) in idiopathic HP patients were significantly enlarged compared to the healthy control group. Based on its pathology, we hypothesize that the fibrosis and inflammatory lymphoplasmacytic infiltrated in EOM potentially involves microstructural changes, leading to structural volume alterations in idiopathic HP patients. This is supported by studies conducted in IgG4-related disease (RD), which is a subset of HP that associated with an autoimmune disorder, characterized by lymphoplasmacytic infiltrate with predominance of IgG4-positive plasma cell and the most commonly affected organ are the orbits included EOM causing EOM enlargements [11–13].

The predilection of muscle involvement in idiopathic HP remains unclear. In our study, we observed that the left inferior rectus and both medial rectus muscles were significantly larger. As compared with other diseases causing multiple EOM enlargements, such as IgG4-related disease, the LR muscle is most frequently affected, and in Graves’ ophthalmopathy, the IR, MR, SR, and LR are mostly involved in that order [14–17]. However, because our sample size was small, we were unable to conclude the predilection of specific muscles.

Extraocular muscle (EOM) volume may serve as a novel biomarker for early differential diagnosis of idiopathic hypertrophic pachymeningitis. Inflammatory infiltration of the extraocular muscles leads to hypertrophy and subsequent fibrosis. The enlarged, fibrotic muscles compress orbital structures and exhibit impaired contractility and relaxation. These pathophysiological changes result in mechanical restriction of ocular motility, manifesting as gaze limitations. Therefore, this study was conducted to clarify whether the limitation of ocular motility is related to EOM enlargement or not. However, our study found no significant correlation between increased EOM volume and impaired ocular muscle movement in idiopathic HP patients. Future research may establish its association with disease severity, as increased EOM volume could indicate ocular involvement even in the absence of clinical muscle dysfunction. As compared to Graves’ ophthalmopathy (GO), the most common inflammatory disease, affecting the orbital muscles, which the primary histopathological features are also characterized by interstitial inflammatory edema with cellular infiltration composed of plasma cells, lymphocytes, macrophages, and mast cells [6]. A rough comparison between EOM volume of GO is greater than in idiopathic HP. For example, the mean medial rectus muscle volume in GO is 905 mm³ [18] compared to 560 mm³ in our study. Anyhow, no prior literature directly compared EOM volumes between these two diseases. Future comparative studies would allow for a more precise quantification of these differences. As concluded in GO, the enlarged EOM itself is a significant mechanical impediment. Swollen EOMs physically occupy more space within the orbit, restricting their ability to move freely leading to direct mechanical compression and restriction of ocular movement [9,19]. While in idiopathic HP, clinical manifestations depends on the location of dura infiltration and compression adjacent cranial nerves [9,20]. Specifically, deficits in cranial nerves III, IV, and VI are associated with thickened meninges from the cavernous sinus to the superior orbital fissure [20]. Therefore, we imply that the limitation of EOM in patients with idiopathic HP is caused by cranial nerve compression from the thickened dura rather than muscle enlargement. Further study would provide more information to examine the correlation with ocular motility function or other ophthalmologic sequelae for anticipated useful prognostic data in the therapy of idiopathic HP patient.

There are several limitations to this study. Firstly, due to the rarity of idiopathic HP, the single-center study had a small sample size which limits the generalizability of the findings and may restrict the ability to detect true differences and could slead to statistical inaccuracies. Secondly, being a retrospective study may introduce selection bias and missing data. However, we clarify that we included all available cases of this rare disease within the study period to minimize selection bias and ensured the completeness of the dataset by thoroughly verifying and cross-checking the medical records. Thirdly, although the control group had an overall physical examination and no history of eye symptoms, they did not undergo a complete ophthalmic examination. Next, assessment of extraocular muscle (EOM) motility using cardinal positions of gaze provided only qualitative data. However, that is standardized and commonly used in strabismus literature. Future research would benefit from incorporating quantitative measurement tools such as the Hess screen test and Lancaster screen test to obtain objective measurements. Lastly, this study did not provide volumetric measurement of EOM after treatment interventions which limit the understanding of the dynamics of muscle volume changes in response to treatment. Further prospective cohort study would provide more information to examine the correlation with ocular motility function or other ophthalmologic sequelae for anticipated useful prognostic data in the therapy of idiopathic HP patients.

## 6. Conclusion

In idiopathic HP, patients have significantly larger EOM volumes, particularly in the left IR and both MR muscles. This could be due to the diffuse infiltrative histopathology that potentially involves microstructural changes in the EOM. However, no significant correlation was found between the size of these enlarged EOMs and the extraocular movement limitation.
